# Photocatalytic Removal of Metronidazole Antibiotics from Water Using Novel Ag-N-SnO_2_ Nanohybrid Material

**DOI:** 10.3390/toxics12010036

**Published:** 2024-01-02

**Authors:** Md. Shahriar Hossain Shuvo, Rupna Akther Putul, Khandker Saadat Hossain, Shah Md. Masum, Md. Ashraful Islam Molla

**Affiliations:** 1 Department of Applied Chemistry and Chemical Engineering, Faculty of Engineering and Technology, University of Dhaka, Dhaka 1000, Bangladesh; s-2016214455@aace.du.ac.bd (M.S.H.S.); s-2016814459@acce.du.ac.bd (R.A.P.); 2 Nanophysics and Soft Matter Laboratory, Department of Physics, Faculty of Science, University of Dhaka, Dhaka 1000, Bangladesh; k.s.hossain@du.ac.bd

**Keywords:** photocatalytic degradation, metronidazole, Ag-N-SnO_2_, sunlight, nanohybrid material

## Abstract

In this study, we employed a straightforward synthetic approach using the sol-gel method to fabricate a novel photocatalyst, Ag and N co-doped SnO_2_ (Ag-N-SnO_2_). The synthesized photocatalysts underwent characterization through various techniques including XRD, FTIR, FESEM-EDS, TEM, UV-vis DRS, BET, and XPS. The UV-vis DRS results confirmed a reduction in the bandgap energy of Ag-N-SnO_2_, leading to enhanced absorption of visible light. Additionally, TEM data demonstrated a smaller particle size for Ag-N-SnO_2_, and BET analysis revealed a significant increase in surface area compared to SnO_2_.The efficiency of the Ag-N-SnO_2_ photocatalyst in degrading metronidazole (MNZ) under natural sunlight surpassed that of SnO_2_. Under optimal conditions (Ag-N-SnO_2_ concentration of 0.4 g/L, MNZ concentration of 10 mg/L, pH 9, and 120 min of operation), the highest MNZ photocatalytic removal reached 97.03%. The reaction kinetics followed pseudo-first-order kinetics with a rate constant of 0.026 min^−1^. Investigation into the mineralization of MNZ indicated a substantial decrease in total organic carbon (TOC) values, reaching around 56% in 3 h of sunlight exposure. To elucidate the photocatalytic degradation mechanism of MNZ with Ag-N-SnO_2_, a scavenger test was employed which revealed the dominant role of ^•^O_2_^–^. The results demonstrated the reusability of Ag-N-SnO_2_ for up to four cycles, highlighting its cost-effectiveness and environmental friendliness as a photocatalyst.

## 1. Introduction

Water pollution is a pressing global concern, with a particular focus on the existence of diverse organic pollutants in water sources, notably pharmaceuticals such as antibiotics. In Bangladesh, rivers have been found to contain antibiotics from sources like aquaculture, veterinary use, and human consumption [1]. The poultry industry employs antibiotics like MNZ for veterinary purposes, and poultry waste is repurposed in the production of fish feed [1,2]. This escalating use of antibiotics has detrimental consequences, including the development of antimicrobial resistance in humans and disruption of aquatic ecosystems [2,3]. MNZ, a prominent antibiotic globally and part of the nitroimidazole class, is among the top ten most commonly prescribed medications, even during pregnancy [4]. The presence of MNZ in the Brahmaputra River in Bangladesh has concentrations ranging from 0.05 to 13.51 ng/L [1]. Due to its chemical structure, MDG is difficult to fully eliminate using conventional methods [3]. Consequently, a number of techniques were used to eliminate MNZ from water, including adsorption [5], the ozonation process [6], biosorbents [7], solar photoelectro-Fenton [8], and heterogeneous photocatalysis [2]. To achieve the full mineralization of the pharmaceutical waste into CO_2_, H_2_O, and N_2_, an advanced oxidation process (AOP), particularly heterogeneous photocatalysis, is a potential technique for treating wastewater that contains MNZ [2,9]. AOP involves the use of visible and UV light to form a pair of electrons and holes (e^−^/h^+^) at the photocatalyst surface, which results in the formation of hydroxyl radicals (^•^OH) [10]. 

To date, various semiconductors have been identified as suitable candidates for photocatalysis, e.g., TiO_2_, ZnO, SnO_2_, CdS, BiVO_4_, g-C_3_N_4_, and WO_3_ [10,11]. Among these semiconductors, SnO_2_ has captured the interest of researchers owing to its advantageous features such as high oxidation potential, chemical inertness, resistance to corrosion, durability, non-toxicity, cost-effectiveness, and environmentally friendly attributes [12]. Nevertheless, SnO_2_ has significant drawbacks, including a broad bandgap of approximately 3.6 eV, limiting its light absorption to ultraviolet wavelengths and resulting in inefficient use of sunlight. Another significant problem is the quick recombination of photogenerated electrons and holes [13]. To address these limitations, various strategies, such as doping with metals/nonmetals, adjusting the energy band, creating composite photocatalysts, and controlling the material′s morphology, have been employed to design innovative photocatalysts based on SnO_2_ [14,15]. 

Doping involves the alteration of a photocatalyst by introducing impurities, which serve to change its band gap. Metal/nonmetal doping is a component of band gap engineering, which assists in preventing the recombination of electrons by promoting their capture [16]. The incorporation of dopants into the photocatalyst delivers outstanding performance by narrowing the bandgap between the conduction and valence bands, delaying the recombination of e^−^/h^+^ pairs, expanding the surface area, and increasing the pore size of the photocatalysts [15,17]. Among transition or noble metals, Ag is the least expensive noble metal. Again, the Ag dopant functions as an electron sink, successfully capturing photoexcited electrons from SnO_2_, preventing the recombination of e^−^/h^+^ pairs [9]. For instance, Ag-doped SnO_2_ was demonstrated to achieve impressive degradation rates, including an 87% reduction in carbamazepine within 120 min [18]. Furthermore, nitrogen (N) is one of the nonmetal dopants that effectively enhances semiconductor oxides′ absorption of light [15]. Bhawna et al. reported that N-SnO_2_ nanoparticles displayed significant photodegradation rates of methylene blue (93%) and methyl orange (83%) dyes under UV light exposure [19]. Siddique et al. synthesized Ag/Bi/SnO_2_ nanohybrid material using green chemistry route and documented photodegradation of methylene blue [20]. Moreover, Bashiri et al. prepared a Fe_3_O_4_/rGO-TiO_2_ photocatalyst using the hydrothermal method to remove MNZ from water [3]. Recently, Fahim et al. synthesized B-Sn/TiO_2_ nanoparticles and investigated the photodegradation of MNZ with UV-C and natural sunlight irradiation [2]. 

Herein, SnO_2_ and Ag-SnO_2_, N-SnO_2_, and Ag-N-SnO_2_ photocatalysts were synthesized by the sol-gel method. Both the synthesized Ag-N-SnO_2_ and the degradation of MNZ using this photocatalyst under natural solar radiation are innovative since there has not been any study published to date. The study also delved into understanding the potential mechanism involved in the photocatalytic breakdown of MNZ and assessed the reusability of the photocatalyst over four cycles. Furthermore, the goal of the research was to identify the optimal conditions necessary for achieving the maximum elimination of MNZ from water, with a view to its potential application in treating pharmaceutical wastewater.

## 2. Materials and Methods

### 2.1. Materials

The materials employed in this research were of reagent-grade quality and had not undergone any earlier purifying procedures. The chemical substances included SnCl_4_·5H_2_O (Merck, Darmstadt, Germany), AgNO_3_ (Merck, Germany), urea (CH_4_N_2_O, Merck, Germany), acetic acid (CH_3_COOH, Merck, Germany) and NaOH pellets and HCl (Active Fine Chemicals Ltd., Dhaka, Bangladesh). Ascorbic acid (C_6_H_8_O_6_), di-ammonium oxalate monohydrate ((NH_4_)_2_C_2_O_4_·H_2_O), and 2-propanol ((CH_3_)_2_CHOH) were also supplied by Merck (Darmstadt, Germany). Metronidazole (C_6_H_9_N_3_O_3_) was sourced from Corden Pharma, Italy. Throughout the experiments, a HITECH laboratory water purification system generated deionized water (DI), which was utilized. 

### 2.2. Synthesis of Photocatalyst

Ag-N-SnO_2_ photocatalysts were synthesized through a sol-gel method, as illustrated in Figure S1. Initially, 4.373 g of 0.2 M tin (IV) chloride pentahydrate was mixed with 22 mL of DI water and agitated for 10 min. To produce the precursor that has N and Ag in it, an aqueous solution of 0.00927 M AgNO_3_ (0.0945 g AgNO_3_ dissolved in 20 mL of DI) and an aqueous solution of 0.0713 M urea (CH_4_N_2_O) (0.257 g urea dissolved in 20 mL of DI) were gradually incorporated into the tin chloride solution while stirring for 10 min. To control the reactions, 2 mL of acetic acid was added initially to control the reactions, and then 25 mL of a 2M NaOH solution was gradually introduced to maintain a pH of 7. The sol solution was continuously stirred for 1 h to ensure homogeneity. The sol was then allowed to mature for 72 h to form a dense gel. After that, the gel was filtered, washed with DI water, and dried for 1 hour at 110 °C. Finally, the material was placed in a calcination chamber for 2 h at 500 °C to obtain the desired Ag-N-SnO_2_ photocatalysts. To produce Ag-SnO_2_, N-SnO_2_, and SnO_2_, the same experimental methodology was implemented, but without the addition of CH_4_N_2_O, AgNO_3_, and both precursors, respectively. 

### 2.3. Characterization

The characteristics of the samples′ XRD spectra in the 10°–70° 2θ range were assessed using an X-ray diffractometer (Ultima IV, Rigaku, Japan) equipped with CuK radiation (wavelength = 0.154 nm, voltage: 40 KV, current: 1.64 mA). The samples′ elemental composition was ascertained by utilizing a field emission scanning electron microscope (FESEM, JSM-7610F, JEOL Ltd., Japan) outfitted with energy-dispersive X-ray spectroscopy (EDS). For a detailed examination of the surface morphology, a transmission electron microscope (TEM, Talos F200X G2, produced in the Czech Republic) was employed. The identification of chemical bonds present in the samples was carried out using an IR Prestige-21 spectrophotometer (Shimadzu, Japan), covering the wavenumber range between 4000 and 400 cm^–1^. The spectrophotometer had a resolution of 5 cm^–1^, and 30 scans were conducted for each measurement. UV–vis diffuse reflectance spectra (DRS) of the samples within the 300–650 nm range were examined using a UV–visible/NIR spectrophotometer UH4150 (Hitachi, Japan). Measurements of zeta potential (ζ) were performed to determine the point of zero charge (pH_PZC_) by employing the Nano PARTICA SZ-100V2 series (HORIBA Scientific Ltd., Japan). The distribution of pore sizes and surface area of the photocatalyst were calculated based on N_2_ adsorption-desorption isotherms obtained using a Brunauer-Emmett-Teller (BET) sorptometer (BET-201-A, PMI, Ithaca, NY, USA). These parameters were examined by employing the BET and Barrett-Joyner-Halenda (BJH) techniques. For X-ray photoelectron spectroscopy (XPS) analysis, an Al Kα monochromatic X-ray generator (wavelength: 1486.69 eV, voltage: 15 kV, current: 10 mA) was used in conjunction with a Thermo Scientific photoelectron spectrometer (UK).

### 2.4. Studying Photocatalytic Activity

The evaluation of MNZ removal efficiency using SnO_2_, Ag-SnO_2_, N-SnO_2_, and Ag-N-SnO_2_ photocatalysts involved varying operational parameters, including pH, dosage, irradiation duration, and initial MNZ concentration. A standard MNZ solution with a characteristic absorption peak at 319 nm [2] (Figure S2) was used for natural solar irradiation in an open-air environment. In a typical experiment, 50 mL of the standard MNZ solution was placed in a 250 mL beaker, and 20 mg of the fabricated photocatalyst was added, as demonstrated in Figure S3. Subsequently, under sunlight exposure, 3 mL of the MNZ solution was periodically collected and filtered using a 0.45 µm Advantec membrane filter. The residual MNZ concentration after degradation under different conditions was identified by measuring the absorbance with a UV-1700 spectrophotometer (Shimadzu, Japan). 

All batch experiments were executed under consistent conditions, with coordinates of 23.7275° N and 90.4019° E, solar intensity at approximately 3.4 mW/cm^2^, temperature around 35 °C, and the time of year being July to August. Experiments were carried out on sunny days between 11:00 and 14:00 BST (Bangladesh standard time) to determine the percentage of MNZ elimination using the fabricated photocatalysts. Before degradation studies, the solutions′ pH was changed using diluted HCl (0.1 M) and NaOH (0.1 M). The initial pH of the MNZ solution was 5.8 [2]. The efficiency of MNZ removal was enumerated using the formula below:

(1) $$\mathrm{MNZ}\;\mathrm{removal}\;\mathrm{efficiency}\;(\%)=\frac{C_{0}-C}{C_{0}}\times 100$$

where *C*_0_ and *C* stand for the initial and final MNZ concentrations. 

A Shimadzu TOC analyzer (TOC–V CPH, Shimadzu, Japan) was utilized to measure the total organic carbon (TOC). Each experiment was conducted in triplicate, and the average values of the results were reported. The range of the relative standard deviations was 2% to 10%. In order to investigate the reactive species that take part in the process of photodegradation, three distinct chemical scavengers—ascorbic acid (AA), 2-propanol, and di-ammonium oxalate monohydrate (AO) were employed. AA functioned as a scavenger of superoxide (^•^O_2_^–^), 2-propanol as a scavenger of hydroxyl radical (^•^OH), and AO as a scavenger of holes (h^+^) [2].

## 3. Results and Discussion

### 3.1. Morphology and Structure of Photocatalysts

**Figure 1:**
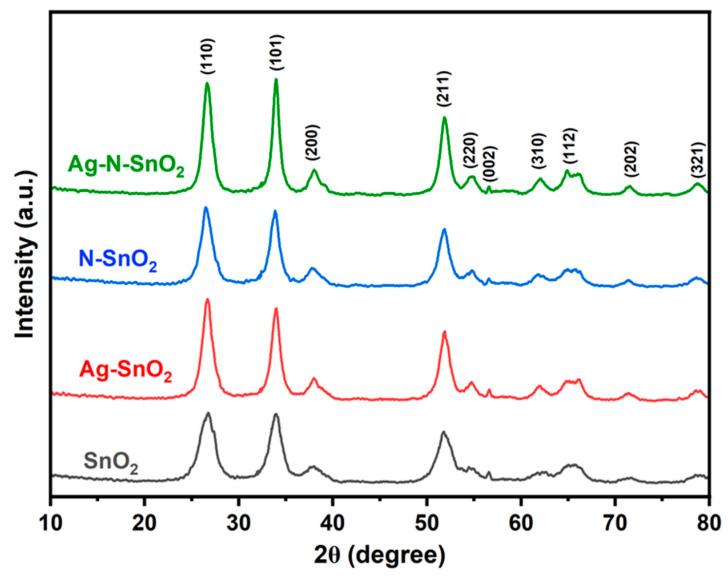
XRD patterns of SnO_2_, Ag-SnO_2_, N-SnO_2_, and Ag-N-SnO_2_.

Clearly defined reflections at 2θ values of 26.7, 34.1, 38.1, 52.0, 54.8, 57.9, 62.0, 64.8, 66.1, 71.4, and 78.8 are found in the SnO_2_ XRD pattern (Figure 1). These reflections are associated with the (110), (101), (200), (211), (220), (002), (310), (112), (301), (202), and (321) planes of the tetragonal rutile SnO_2_ structure (JCPDS No: 41-1445) [21]. However, similar XRD patterns with the above mentioned characteristic peaks were obtained for Ag-SnO_2_, N-SnO_2_ and Ag-N-SnO_2_. The absence of characteristic peaks in the Ag and N phases indicates that doping does not change the crystal structure. Moreover, the reason is the high elemental dispersion or the low concentration of either doping element [22,23]. The related materials′ average crystallite sizes were determined using the Debye-Scherrer formula [19] and are presented in Table 1. From this, the estimated average sizes of the crystallite were 6.27 nm, 7.67 nm, 7.78 nm, and 6.92 nm for SnO_2_, Ag-SnO_2_, N-SnO_2_, and Ag-N-SnO_2_, respectively [19,20]. The slope (*η*) of the Williamson–Hall (W-H) plot of *β*cos*θ* versus 4sin*θ* was used to calculate the crystal strains of SnO_2_, Ag-SnO_2_, N-SnO_2_, and Ag-N-SnO_2_ [11]. Tensile strain for crystal SnO_2_ was detected for Ag-SnO_2_, N-SnO_2_, and Ag-N-SnO_2_, as indicated by positive slopes of 0.0058, 0.0064, and 0.0073, respectively, as illustrated in Figure S4.

**Figure 2:**
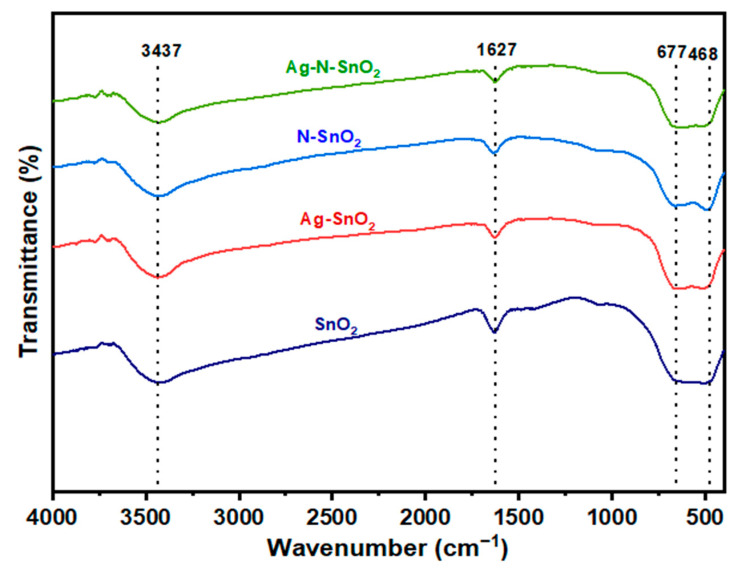
FTIR spectra of SnO_2_, Ag-SnO_2_, N-SnO_2_, and Ag-N-SnO_2_.

The existence of various functional groups is studied using Fourier transform infrared spectroscopy (FTIR) in the frequency range 4000 cm^−1^ to 400 cm^–1^. The FTIR transmission spectra of photocatalysts are given in Figure 2. The presence of hydroxyl groups is indicated by the absorption band at 3437 cm^–1^, which is explained by the stretching vibration of the O–H, while the bending vibration of the O–H bond is responsible for the band at 1649 cm^–1^. This is because SnO_2_ retained certain adsorbed water [24]. The 400–680 cm^–1^ range is linked to the stretching vibrations of bridged Sn–O–Sn and Ag–O–Sn bonds. [23]. Also, the peak at 468 cm^–1^ represents the asymmetric vibrations of Sn–N–Sn [21]. Furthermore, the introduction of the dopants into the SnO_2_ crystal structure is responsible for the minor alterations in band locations and intensities. 

**Figure 3:**
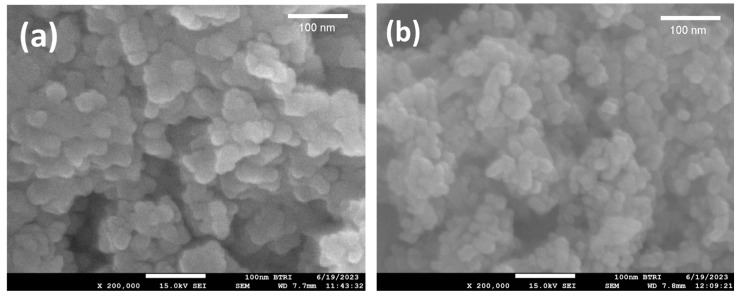

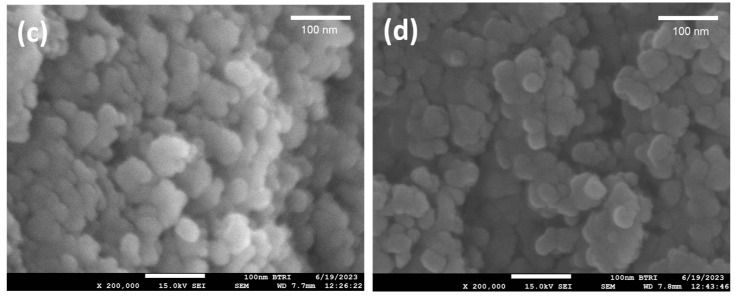
FESEM images of (**a**) SnO_2_, (**b**) Ag-SnO_2_, (**c**) N-SnO_2_, and (**d**) Ag-N-SnO_2_.

The surface and particle morphologies of the SnO_2_, Ag-SnO_2_, N-SnO_2_, and Ag-N-SnO_2_ photocatalysts assessed by FESEM are visualized in Figure 3. FESEM is carried out at a magnification of 200,000×. As shown in Figure 3a, SnO_2_ displays uniformly distributed, globular-shaped, tiny nanoparticles [25]. The micrograph of Ag-SnO_2_ demonstrates a lower spherical-shaped particle with a lower degree of agglomeration, resulting in a reduction in the grain size of the particles (Figure 3b). The micrograph of N-SnO_2_ illustrates slightly larger spherical-shaped particles with a small degree of agglomeration, as displayed in Figure 3c. The micrograph of Ag-N-SnO_2_ demonstrates that the fine nanoparticles that are spherical in shape have a homogeneous distribution with a higher aggregation (Figure 3d). It is observed that Ag doping increases the extent of agglomeration but helps to decrease the particle size, whereas N doping lowers the percentage of agglomeration but increases the particle size [24,26]. To determine the average particle size of SnO_2_, Ag-SnO_2_, N-SnO_2_, and Ag-N-SnO_2_ ImageJ software (version: 1.54) was utilized, the photocatalysts were marked at 60, 55, 66, and 73 different locations and represented as a size distribution histogram to the log-normal distribution curve (Figure S5). The average particle sizes of SnO_2_, Ag-SnO_2_, N-SnO_2_, and Ag-N-SnO_2_ were found to be 30.04 nm, 16.33 nm, 25.15 nm, and 18.89 nm, respectively. 

**Figure 4:**
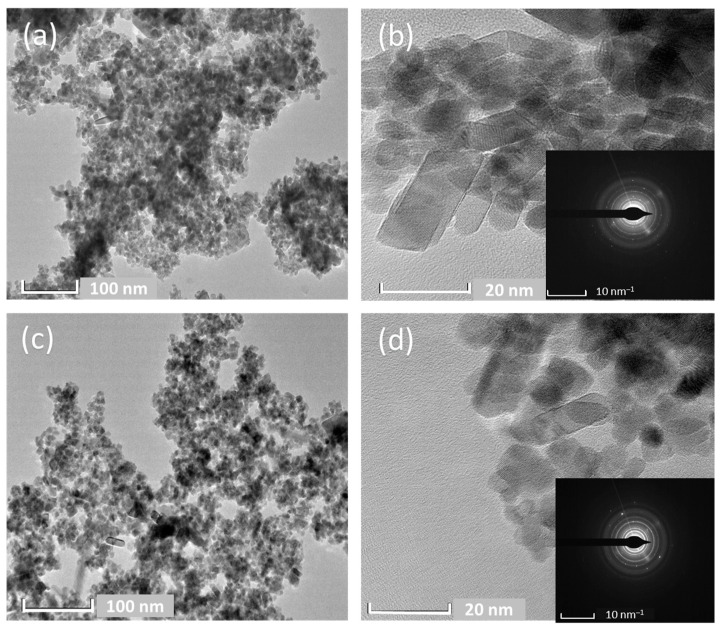
TEM, HRTEM, and SAED patterns (inset) of (**a**,**b**) SnO_2_ and (**c**,**d**) Ag-N-SnO_2_.

To examine the surface and microstructure of the SnO_2_ and Ag-N-SnO_2_ photocatalysts, TEM and high-resolution TEM (HRTEM) observations were performed, as illustrated in Figure 4. Aggregated spherical-shaped particles, with average particle sizes from 8 to 19 nm for SnO_2_ and 4 to 17 nm for Ag-N-SnO_2_, are visible the TEM micrographs (Figure 4a,c). The introduction of both Ag and N led to a reduction in the particle size of SnO_2_, a phenomenon also observed in field-emission scanning electron microscopy (FESEM) analyses. Figure 4b,d provide HRTEM images of SnO_2_ and Ag-N-SnO_2_, respectively. Additionally, inset images in Figure 4b,d showing selected area diffraction patterns (SAED) reveal the presence of continuous ring-like patterns, confirming the polycrystalline nature of the SnO_2_ and Ag-N-SnO_2_ photocatalysts [27].

Figure S6 displays the line-scanning outputs of SnO_2_, Ag-SnO_2_, N-SnO_2_, and Ag-N-SnO_2_ photocatalysts using EDS. Sharp peaks of elemental Sn and O are seen from the EDS spectrum of SnO_2_ (Figure S6a). The prominent peaks of elemental Ag, Sn, and O in the Ag-SnO_2_ EDS spectra are displayed in Figure S6b. In the EDS spectra of N-SnO_2_, elements N, Sn, and O are represented as peaks (Figure S6c). The presence of all four elements were confirmed by the discovery of peaks associated with the elements Ag, N, Sn, and O from the Ag-N-SnO_2_ EDS spectra study displayed in Figure S6d. Figure S6 shows Na and Cl as impurities in the composition of all the photocatalysts. These impurities may occur from the reaction precursors during synthesis [20]. Table S1 displays the elemental composition of each of the four photocatalysts. The mass and atom percentages that were found are extremely near to stoichiometric values. 

**Figure 5:**
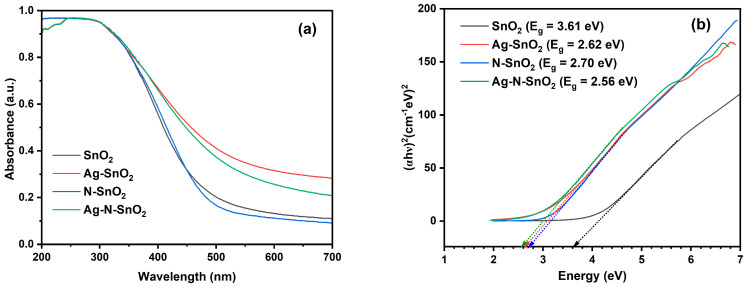
(**a**) UV−vis DRS spectra and (**b**) Tauc plots of SnO_2_, Ag-SnO_2_, N-SnO_2_, and Ag-N-SnO_2_.

Diffuse reflectance spectroscopy (DRS) was used on photocatalysts from 200 nm to 700 nm to investigate the optical behavior as displayed in Figure 5. The band gap energy (*E*_g_) can be obtained from the Tauc equation by extrapolating the linear slope from the plot of (*αhν*)^2^ on the ordinate and *hν* (the photon energy) on the abscissa [28]. Table 1 presents the band gap energies of SnO_2_, Ag-SnO_2_, N-SnO_2_, and Ag-N-SnO_2_. In Figure 5a, no absorption was detected in the visible region for SnO_2_. However, there was a notable increase in absorption in the UV range, specifically between 260 and 370 nm, indicating an estimated band gap energy of approximately 3.61 eV [29]. However, Ag-SnO_2_ exhibited significant absorption in the UV range of 240–350 nm and weak absorption in the visible region of 400–650 nm, which is associated with the band gap of Ag-SnO_2_ at about 2.62 eV (Figure 5a,b). As seen in Figure 5a,b, N-SnO_2_ showed a little shift towards a higher wavelength with a bandgap of 2.70 eV. Due to N doping, part of the O atoms in the SnO_2_ lattice are replaced with N atoms. As a result, the absorption edge shifts to a lower energy because the N 2p orbitals overlapped with the O 2p orbitals in the valence band [19]. Figure 5b shows that the estimated band gap energy for Ag-N-SnO_2_ is 2.56 eV. Hence, the incorporation of both Ag and N reduces the band gap energy of Ag-N-SnO_2_, which is lower than that of SnO_2_.

**Figure 6:**
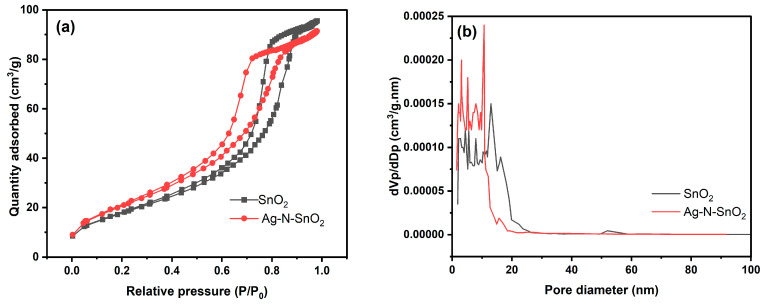
(**a**) N_2_ isotherms for adsorption-desorption and (**b**) BJH curves of SnO_2_ and Ag-N-SnO_2_.

Figure 6a displays the nitrogen adsorption-desorption isotherms of the SnO_2_ and Ag-N-SnO_2_ photocatalysts. The IUPAC classifies the isotherms, which have H3 hysteresis loops, as type IV. The BJH pore size distributions for SnO_2_ and Ag-N-SnO_2_ are illustrated in Figure 6b. Table 1 presents the BET parameters of SnO_2_ and Ag-N-SnO_2_. This table indicates that Ag-N-SnO_2_ has a BET surface area of 78.24 m^2^/g, an average pore diameter of 7.195 nm, and a total pore volume of 0.1407 cm^3^/g. On the other hand, the BET surface area of SnO_2_ is 66.66 m^2^/g, with a total pore volume of 0.1472 cm^3^/g and an average pore diameter of 8.834 nm. The Ag-N-SnO_2_ photocatalyst was found to have a greater surface area than SnO_2_ because the introduction of Ag and N into the crystal structure had a notable impact, causing the photocatalyst to create additional active sites [20].

**Figure 7:**
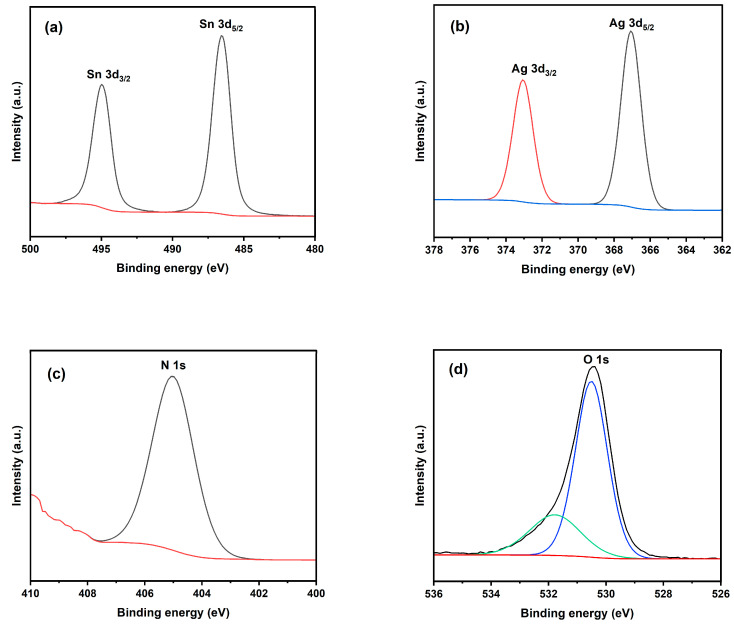
XPS spectra of (**a**) Sn 3d, (**b**) Ag 3d, (**c**) N 1s and (**d**) O 1s.

The SnO_2_ and Ag-N-SnO_2_ photocatalysts′ surface compositions and associated valence states were investigated using X-ray photoelectron spectroscopy (XPS). The XPS full survey spectrum of SnO_2_ is displayed in Figure S7a, where the peaks of the Sn, O, and C components are clearly visible. Figure S7b shows the XPS full survey spectrum of Ag-N-SnO_2_ which distinctly indicates the peaks of the Sn, Ag, N, O, and C elements. The presence of atmospheric CO_2_ captured throughout the measures for characterization could be the cause of the C element observed in the XPS spectrum [30]. The XPS spectra are used to determine the atomic percentage and binding energies of the various elements in Ag-N-SnO_2_, as presented in Table S2. As shown in Figure 7a, the XPS peaks positioned at 487 and 495 eV are related to Sn 3d_5/2_ and Sn 3d_3/2_, pointing out the presence of Sn^4+^ in SnO_2_ and Ag-N-SnO_2_. Additionally, at 368 eV and 373 eV, the two energy peaks are observed (Figure 7b), which are related to Ag 3d_5/2_ and Ag 3d_3/2_ for Ag^0^ [9,23]. The binding energy centered at 413 eV in the deconvoluted core-level N 1s XPS spectrum (Figure 7c) indicates that N^3–^ is present in the lattice and can be attributed to the Sn-O-N linkage [19]. In the deconvoluted O 1s spectra (Figure 7d), the peak at 531.8 eV represents oxygen bound to C as C=O or C-O, whereas the other peak at 530.4 eV represent the lattice O^2–^ [10].

### 3.2. Photocatalytic Activity

**Figure 8:**
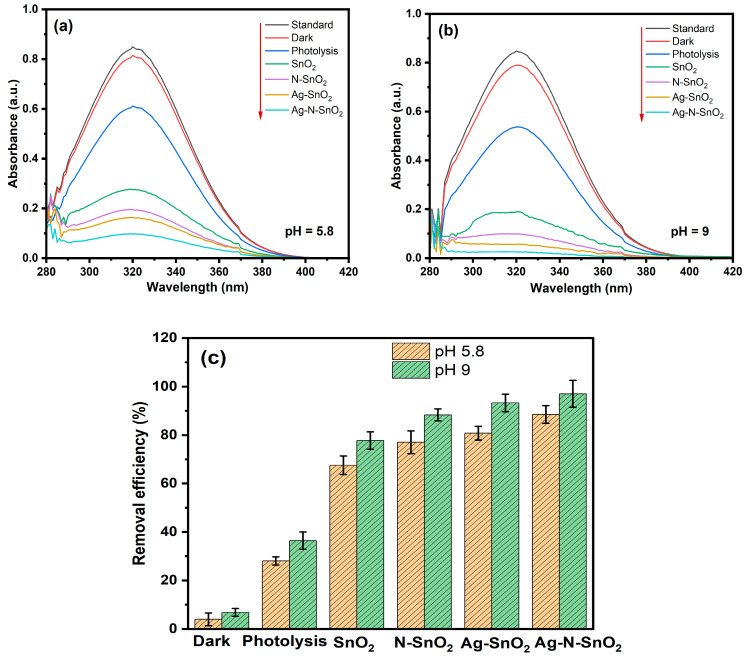
(**a**,**b**) Sunlight irradiation spectrum results demonstrating the decrease in the intensity of MNZ solution and (**c**) photocatalytic degradation, adsorption in darkness, and photolysis for MNZ.

The UV-visible absorption spectra of MNZ with SnO_2_, Ag-SnO_2_, N-SnO_2_, and Ag-N-SnO_2_ photocatalysts are demonstrated in Figure 8a (natural pH 5.8) and Figure 8b (optimized pH 9). The MNZ major peak′s intensity (*λ*_max_ = 319 nm) decreases with time under various photocatalysts. The photocatalytic MNZ degradation in the presence of photocatalysts and solar irradiation causes the peak intensity to decrease. MNZ was subjected to photolysis for 120 min without the use of a photocatalyst, at a natural pH of 5.8 and an optimum pH of 9. At pH 5.8 and pH 9, the photolysis of the MNZ solution is found to be 28.05% and 36.42%, respectively. Another set of tests was performed in the absence of sunlight to ascertain the effectiveness of MNZ elimination caused by adsorption on the Ag-N-SnO_2_ photocatalyst surface. At pH 5.8 and pH 9, the removal efficiency of MNZ with Ag-N-SnO_2_ in the dark for 120 min is found to be 4.00% and 6.82%, respectively. It is found that for both pH values, MNZ adsorption on Ag-N-SnO_2_ is minimal [3]. As demonstrated in Figure 8c, a number of tests were carried out using SnO_2_, Ag-SnO_2_, N-SnO_2_, and Ag-N-SnO_2_ photocatalysts at natural pH 5.8 and optimized pH 9 maintaining other parameters persistent. After 120 min of exposure to sunlight, the results of the photocatalytic experiments indicate that under the initial pH conditions of 5.8, the removal efficiency of MNZ was 67.58% for SnO_2_, 77.03% for N-SnO_2_, 80.81% for Ag-SnO_2_, and 88.54% for Ag-N-SnO_2_. However, when the pH was optimized to 9, the MNZ removal efficiencies improved to 77.70% for SnO_2_, 88.34% for N-SnO_2_, 93.26% for Ag-SnO_2_, and 97.03% for Ag-N-SnO_2_. Thus, Ag-N-SnO_2_ demonstrated superior photocatalytic performance compared to SnO_2_ because of its reduced band gap energy, greater surface area, and smaller particle size, as supported by the literature reviews presented in Table 2 [31,32,33,34].

### 3.3. Operational Parameters Optimization

**Figure 9:**
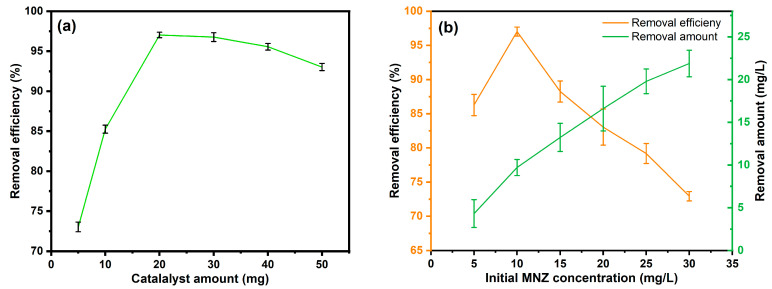

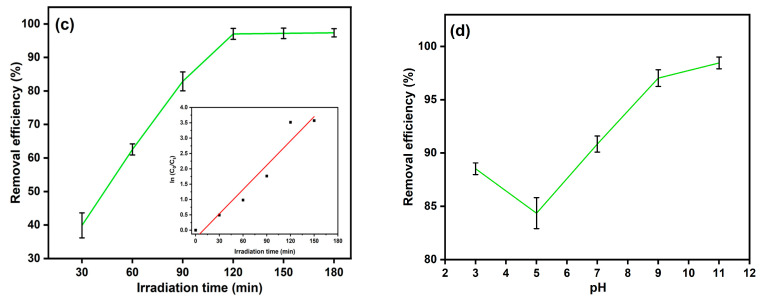
(**a**) Effect of photocatalyst dosage (**b**) effect of initial MNZ concentration (**c**) effect of time and plot of ln(*C*_0_/*C*_t_) versus irradiation time (inset) and (**d**) effect of solution pH on the photocatalytic MNZ degradation with Ag-N-SnO_2_.

Experiments were carried out using the most active photocatalyst, Ag-N-SnO_2_, altering the amount, ranging from 5 to 50 mg, to investigate how photocatalyst dose affects MNZ photocatalytic degradation (Figure 9a). It was observed that when photocatalyst loading was raised from 5 to 20 mg, the MNZ removal effectiveness increased linearly from 73.02% to 97.03%. When the photocatalyst amount was increased up to 30 and 50 mg, respectively, the removal efficiency did not rise any further and instead decreased to 96.76% and 93.02%, respectively. This implies that a dosage of 20 mg is the maximum for MNZ degradation. The increase in removal effectiveness with increased catalyst quantity may be explained by the photocatalyst′s greater active site availability compared to the MNZ molecules present. Because only a small number of active radicals are generated at low photocatalyst doses, there are not enough catalyst active sites to support larger concentrations of MNZ molecules [35]. A high loading of photocatalyst can lead to the scattering of light photons and block sunlight penetration due to increased turbidity. This phenomenon results in a decrease in the efficiency of photocatalytic removal [36,37]. Additionally, an excessive amount of catalyst can lead to the agglomeration of catalyst particles, reducing the exposed surface area and rendering the catalyst inactive. Therefore, the use of 20 mg of Ag-N-SnO_2_ photocatalyst is chosen as the optimal amount for investigating various parameters in the photocatalytic MNZ degradation.

A gradual change in the initial MNZ solution concentration from 5 mg/L to 30 mg/L was used to study the effect on the photodegradation efficiency (Figure 9b). It was observed that on increasing the concentration of MNZ, the degradation efficiency increased from 86.28% to 97.03% for 10 mg/L. But a further increase in concentration resulted in decreased degradation efficiency to 72.93% for 30 mg/L. The amount of MNZ removal was calculated for each sample, and it increases with increasing initial concentrations of MNZ. Maximum degradation efficiency relative to the initial MNZ concentration is close to 10 mg/L [38]. This effect can be elucidated by the fact that the number of active radicals in a sample decreases with concentration, making it insufficient to degrade a greater number of molecules. Furthermore, the presence of more molecules obstructs light, causing photons to be cut off before they reach the catalyst′s active surface and lowering photodegradation efficiency [39]. So, the 10 mg/L MNZ initial concentration is optimized to examine other parameters.

Figure 9c depicts the degradation efficiency of MNZ as a function of irradiation time, and it is evident that the effectiveness of MNZ removal increased with the prolongation of irradiation time under the optimal conditions of sunlight exposure. It is observed that almost 40% degradation occurs within 30 min. The 97.03% MNZ degradation occurs within 120 min of irradiation time and after that the rise of the removal efficiency is not noteworthy due to a great reduction in MNZ molecules in solution [10]. Pseudo-first order reaction kinetics was studied to determine the rate constant of MNZ degradation, which was found to be 0.026 min^−1^ with a correlation coefficient (R^2^) value of 0.94, as demonstrated in the inset image of Figure 9c.

The experiments involved adjusting the pH within the range of 3 to 11 without changing other parameters, aiming to understand the impact of pH as illustrated in Figure 9d. It was observed that MNZ exhibited high stability in the pH range of 3.9 to 6.6, with the highest stability observed at a pH of 5.6 [40]. Consequently, at pH 5, MNZ degradation was at its lowest. In general, when the pH deviates from 5, the efficiency of degradation increases. Notably, at the most alkaline pH of 11, the degradation rate reached 98.46%. The removal efficiency was 97.03% at pH 9, considered the optimal pH due to minimal variation in degradation efficiency between pH 9 and pH 11. The photoactivity of the photocatalyst is directly influenced by the acidity or alkalinity of the solution, which, in turn, affects the surface charge characteristics. The surface charge is the electrical potential difference between the catalyst′s surface and its surrounding medium, and it significantly influences photocatalytic reactions. The presence of hydroxyl groups on the catalyst′s surface is a crucial factor determining this surface charge [41]. 

At pH 3, 5, 7, 9, and 11, the zeta potential of Ag-N-SnO_2_ is measured. Figure S8 demonstrates that pH_PZC_ for Ag-N-SnO_2_ is 5.56. Consequently, at pH<pH_PZC_, the catalyst surface becomes positively charged due to the protonation reaction. Conversely, at pH > pH_PZC_, deprotonation reactions occur, resulting in a negatively charged catalyst surface. In an alkaline environment with a pH of 9, where the catalyst′s surface is negatively charged (pH > pH_PZC_), and MNZ, with pKa values of 2.55 and 14.44, remains protonated, there is an electrostatic attraction between the catalyst and the MNZ molecules, leading to a faster degradation of MNZ [42]. Therefore, an alkaline environment enhances photocatalysis, as it promotes the generation of more hydroxyl radicals from the available OH^–^ in the solution.

### 3.4. MNZ Mineralization

**Figure 10:**
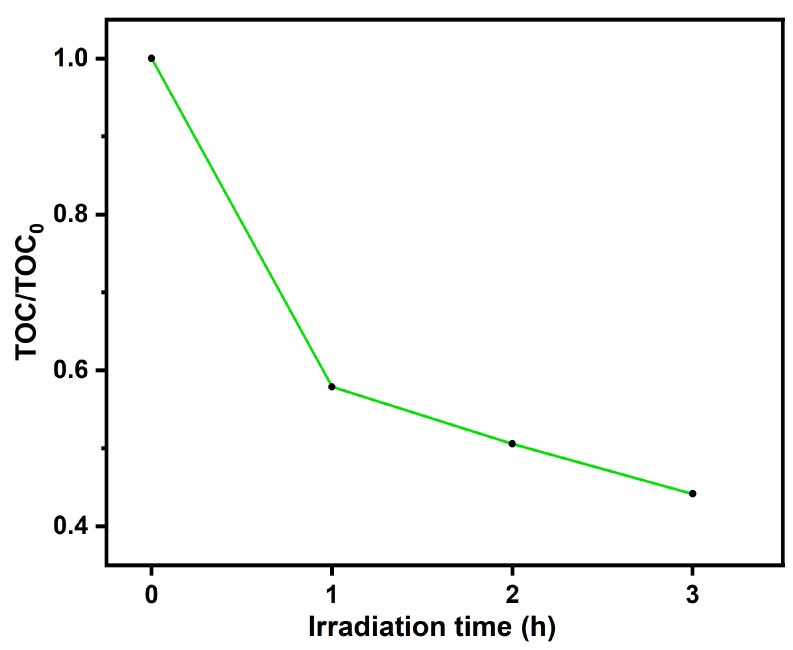
Mineralization of MNZ with Ag-N-SnO_2_ using natural sunlight irradiation.

The total organic carbon (TOC) measurement was carried out to examine the mineralization of MNZ, as shown in Figure 10. The TOC removal values make it clear that after 1 h of irradiation, a considerable amount of organic degradation byproducts remained in solution. The mineralization of MNZ was observed at about 42.11% after 1 h irradiation by sunlight. During 3 h of solar irradiation, the mineralization of MNZ was found to be 55.85%. This suggests that during this initial period, the breakdown of organic molecules resulted in smaller byproducts, while complete mineralization into CO_2_, H_2_O, and other inorganic substances had not yet occurred. To achieve the full mineralization of these remaining organic components, longer irradiation times may be required [17]. 

### 3.5. Radical Scavenges’ Function

**Figure 11:**
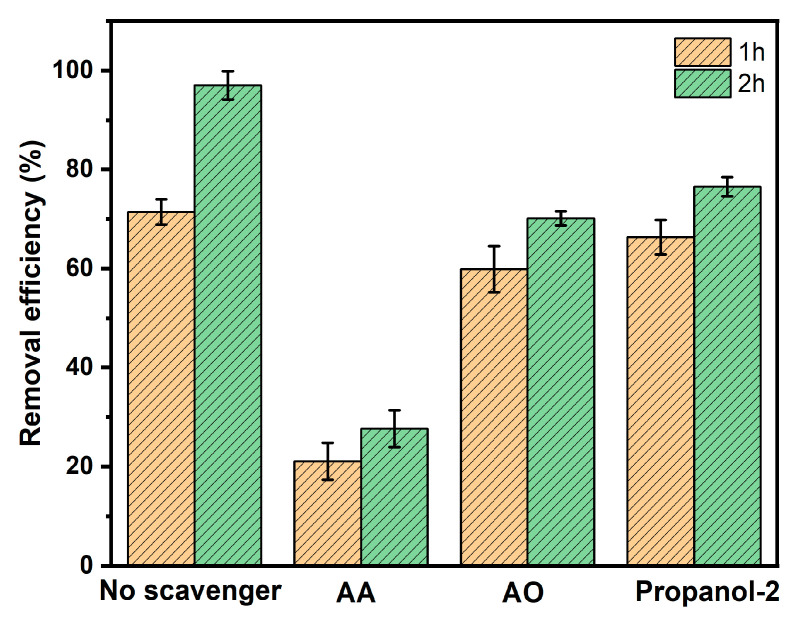
The function of radical scavengers in the photocatalytic MNZ degradation using Ag-N-SnO_2_ under natural sunlight irradiation.

Scavenger tests were conducted under ideal conditions to investigate the role of reactive species in the photocatalytic degradation of MNZ with Ag-N-SnO_2_, as depicted in Figure 11. The scavengers were added to the aqueous MNZ solution prior to the addition of the photocatalyst. Figure 11 illustrates that photocatalytic degradation decreased from 71.44% (without a scavenger) to 66.35% (^•^OH), 59.88% (h^+^), and 21.05% (^•^O_2_^–^), for 1 h upon the addition of 2-propanol, AO and AA as scavengers. Again, without the use of any scavenging agent, the photocatalyst exhibited a significant removal efficiency of 97.03% after 2 h of exposure to solar irradiation. However, with the addition of scavengers, the removal efficiency was reduced to 76.54%, 70.14%, and 27.64%, respectively, after 2 h of sunlight irradiation. So, the degradation of MNZ relied on the presence of ^•^O_2_^–^ radicals to a great extent. The reduction in the degradation of MNZ is a result of active radicals being hindered by different scavengers [2,10]. 

### 3.6. Plausible Degradation Mechanism for MNZ

**Figure 12:**
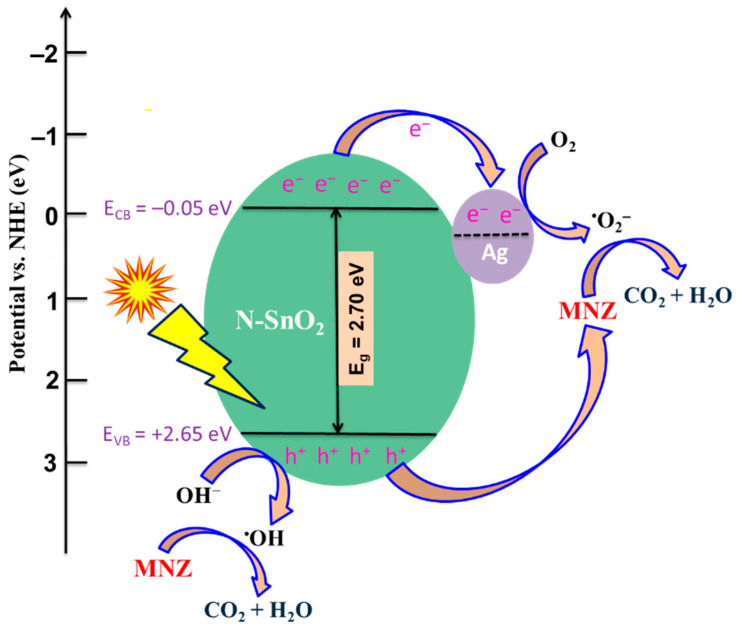
Photocatalytic MNZ degradation mechanism using Ag-N-SnO_2_ under natural sunlight irradiation.

The valence band (VB) value of Ag-N-SnO_2_ was determined by XPS valence spectroscopy, and it was found to be 2.65 eV (Figure S9). The conduction band (CB) values of the sample can be calculated with the following formula: E_CB_ = E_VB_ – E_g_, where E_g_ is the band gap energy [2]. The band gap energy for N-SnO_2_ is 2.70 eV, and its CB value is calculated to be –0.05 eV. The photocatalytic MNZ degradation process under natural sunlight is depicted in Figure 12. The absorption of ultraviolet or visible light results in the elevation of electrons (e^–^) from the VB to the CB of N-SnO_2_. Due to the Fermi energy level of Ag (0.9 eV) being lower than the CB of N-SnO_2_, the most photogenerated e^–^ will transfer from N-SnO_2_ to Ag, while the holes (h^+^) stay in the VB of SnO_2_ [2,9]. These photogenerated e^–^ and h^+^ then engage in a sequence of reactions, giving rise to various radical species such as hydroxyl (^•^OH), superoxide (^•^O_2_^–^), hydroperoxyl (^•^OOH), and consequently other reactive oxygen species (e.g., H_2_O_2_ and OH^–^). Scavenger studies also confirm that ^•^O_2_^–^ is the most active species in the photocatalytic degradation of MNZ molecules. Hydroxyl (^•^OH) radicals also play a pivotal role in the degradation process, as these highly reactive radicals can swiftly attack MNZ in the solution and break them down into CO_2_, H_2_O, and other degradation byproducts [2,18]. To facilitate comprehension, equations 2 to 8 delineate each individual step involved in radical generation and subsequent degradation [2,43].

Ag-N-SnO_2_ + Sunlight (*hν*) → Ag-N-SnO_2_ (e^−^) + Ag-N-SnO_2_ (h^+^) (2)

Ag-N-SnO_2_ (e^−^) + O_2_ → Ag-N-SnO_2_ + ^•^O_2_^−^ (3)

Ag-N-SnO_2_ (h^+^) + H_2_O → Ag-N-SnO_2_ + ^•^OH + H^+^ (4)

H^+^ + ^•^O_2_^−^ → ^•^OOH (5)

^•^OOH + ^•^OOH → H_2_O_2_ + O_2_ (6)

Ag-N-SnO_2_ (e^−^) + H_2_O_2_ → Ag-N-SnO_2_ + ^•^OH + OH^−^ (7)

MNZ + ^•^O_2_^−^/^•^OH → Degradation products → CO_2_ + H_2_O (8)

### 3.7. Ag-N-SnO_2_ Reusability

**Figure 13:**
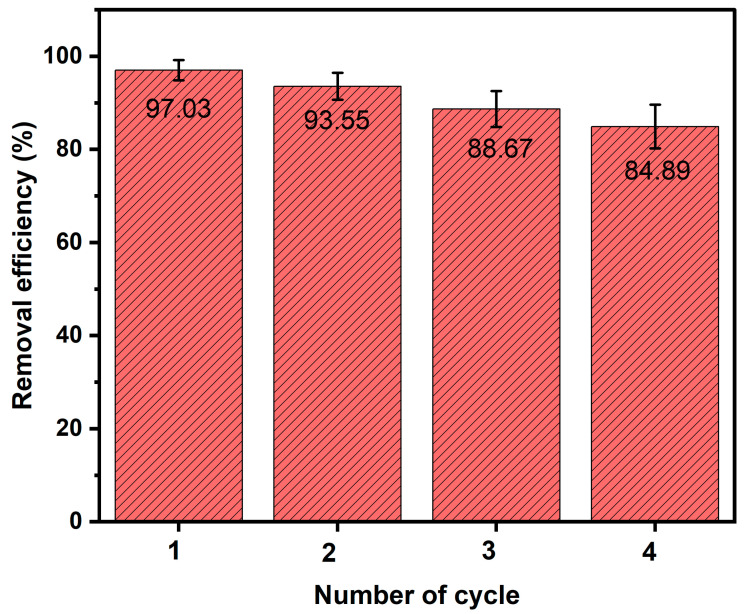
Demonstration of reusability of the Ag-N-SnO_2_ for the photocatalytic MNZ degradation.

Figure 13 depicts the results of reusability experiments on the Ag-N-SnO_2_ photocatalyst. In these experiments, the photocatalyst was subjected to centrifugation, collection, and drying to prepare it for subsequent cycles. The findings revealed a slight reduction in MNZ removal efficiency when using the Ag-N-SnO_2_ photocatalyst in successive cycles, decreasing from 97.03% to 84.89% over four cycles. This decline in removal performance may be attributed to the accumulation of degraded products that inhibit the active sites of the catalyst, potentially obstructing light from reaching the catalyst′s surface. These results indicate that the photocatalyst is well-suited for extended and repeated use in removing similar toxic organic pollutants from aqueous environments. The nearly complete and rapid MNZ removal, combined with its long-term reusability, positions this photocatalyst as a promising option for industrial uses [9].

## 4. Conclusions

The low-cost sol-gel method is used to synthesize SnO_2_, Ag-SnO_2_, N-SnO_2_, and Ag-N-SnO_2_ photocatalysts, followed by a subsequent calcination for 2 h at 500 °C. This unprecedented nanohybrid demonstrates excellent features and capabilities for the photocatalytic removal of MNZ because of the synergistic actions of Ag and N. The synthesized Ag-N-SnO_2_ exhibits an average crystallite size of 6.92 nm and a particle size of about 17 nm. The band gap energy of Ag-N-SnO_2_ is determined to be 2.56 eV. The substantial reduction in band gap energy affected the expansion of optical absorption into the visible light spectrum, which is the major disadvantage of SnO_2_. Hence, the Ag-N-SnO_2_ photocatalyst demonstrates better photocatalytic activity, which was examined by the photocatalytic MNZ degradation using natural sunlight. The MNZ photocatalytic removal efficiency of 97.03% was observed within 120 min. When exposed to sunlight, the TOC values of MNZ declined, suggesting mineralization during the photocatalytic process. Superoxide (^•^O_2_^–^) was a potential active radical for the photocatalytic MNZ degradation. From the photocatalyst reusability study, the catalyst is stable up to four cycles with high removal efficiency. Therefore, the Ag-N-SnO_2_ photocatalyst could serve as a viable substitute for the elimination of dangerous pharmaceutical pollutants.

## Figures and Tables

**Table 1:** Average crystallite size, BET parameters, and band gap energies of photocatalysts.

Photocatalysts	Average Crystallite Size (nm)	BET Parameters			Band Gap Energies (eV)
		Surface Area (m^2^/g)	Pore Volume (cm^3^/g)	Pore Diameter (nm)	
SnO_2_	6.27	66.66	0.1472	8.834	3.61
Ag-SnO_2_	7.67	−	−	−	2.62
N-SnO_2_	7.78	−	−	−	2.70
Ag-N-SnO_2_	6.92	78.24	0.1407	7.195	2.56

**Table 2:** Comparison of Ag-N-SnO_2_′s photocatalytic activity for MNZ elimination with that of the recently published SnO_2_-based photocatalyst.

Photocatalysts	Synthesis Method	Band-Gap Energies (eV)	pH	MNZ Concentration (mg/L)	Photocatalyst Dosage (g/L)	Time (min)	Light Source	Removal Efficiency (%)	Ref.
SnO_2_@TiO_2_/ZrTiO_4_/ZrO_2_	Hydrothermal	3.24	6.5	20	0.07	50	UV light	98	[31]
Fe-doped SnO_2_/Co_3_O_4_	Sol-gel and precipitation	2.30	6	30	2	15	UV light	98	[32]
SnO_2_/MOF-199	-	2.18	3	40	2	240	Sunlight	80	[33]
NiO-SnO_2_/clinoptilolite	Precipitation	2.80	3	2	1.2	180	Sunlight	88	[34]
Ag-N-SnO_2_	Sol-gel	2.56	9	10	0.4	120	Natural sunlight	97	This study
Ag-N-SnO_2_	Sol-gel	2.56	5.8	10	0.4	120	Natural sunlight	88	This study

## Data Availability

Data are contained within the article and Supplementary Materials.

## Supplementary Materials

The following supporting information can be downloaded at: https://www.mdpi.com/article/10.3390/toxics12010036/s1, Figure S1. Block diagram for the synthesis of Ag-N-SnO_2_ photocatalysts; Figure S2. (a) UV-visible spectrum of standard metronidazole (MNZ) solutions at different concentrations and (b) calibration curve of MNZ; Figure S3. Diagram of the photocatalytic MNZ degradation reactor; Figure S4. Williamson-Hall plots of (a) SnO_2_ (b) Ag-SnO_2_ (c) N-SnO_2_ and (d) Ag-N-SnO_2_; Figure S5. Particle size distribution histogram of (a) SnO_2_, (b) Ag-SnO_2_, (c) N-SnO_2_, and (d) Ag-N-SnO_2_, and (e) average particle size of the photocatalysts. Figure S6. EDS pattern of (a) SnO_2_, (b) Ag-SnO_2_, (c) N-SnO_2_, and (d) Ag-N-SnO_2_. Figure S7. XPS survey spectra of (a) SnO_2_ and (b) Ag-N-SnO_2_. Figure S8. Zeta potential measurements of Ag-N-SnO_2_ at different pH values. Figure S9. XPS valence band spectra of Ag-N-SnO_2_. Table S1. Elemental analysis of SnO_2_, Ag-SnO_2_, N-SnO_2_, and Ag-N-SnO_2_ from EDS. Table S2: Atomic percentage and binding energies of different elements of Ag-N-SnO_2_ from XPS.
