# Supplementary material for: Photocatalytic Removal of Metronidazole Antibiotics from Water Using Novel Ag-N-SnO2 Nanohybrid Material
Source: Toxics. 2024 Jan 2;12(1):36. doi: 10.3390/toxics12010036 (PMC10820245; doi:10.3390/toxics12010036)
Supplement: Supplementary file 1 [file toxics-12-00036-s001.zip › toxics-2746396-supplementary.pdf]

toxics

Electronic Supplementary Materials

Photocatalytic Removal of Metronidazole Antibiotics from Water Using Novel Ag-N-SnO_2 Nanohybrid Material

Md. Shahriar Hossain Shuvo [1], Rupna Akther Putul [1], Khandker Saadat Hossain [2], Shah Md. Masum [1,*] and Md. Ashraful Islam Molla [1,*]

[1] Department of Applied Chemistry and Chemical Engineering, Faculty of Engineering and Technology, University of Dhaka, Dhaka 1000, Bangladesh; s-2016214455@aace.du.ac.bd (M.S.H.S.); s-2016814459@acce.du.ac.bd (R.A.P)
[2] Nanophysics and Soft Matter Laboratory, Department of Physics, Faculty of Science, University of Dhaka, Dhaka 1000, Bangladesh; k.s.hossain@du.ac.bd (K.S.H)
* Correspondence: masumacce@du.ac.bd (S.M.M.); ashraful.acce@du.ac.bd (M.A.I.M.); Tel.: +88-017-4946-7276 (S.M.M.); +88-015-5235-9706 (M.A.I.M.)

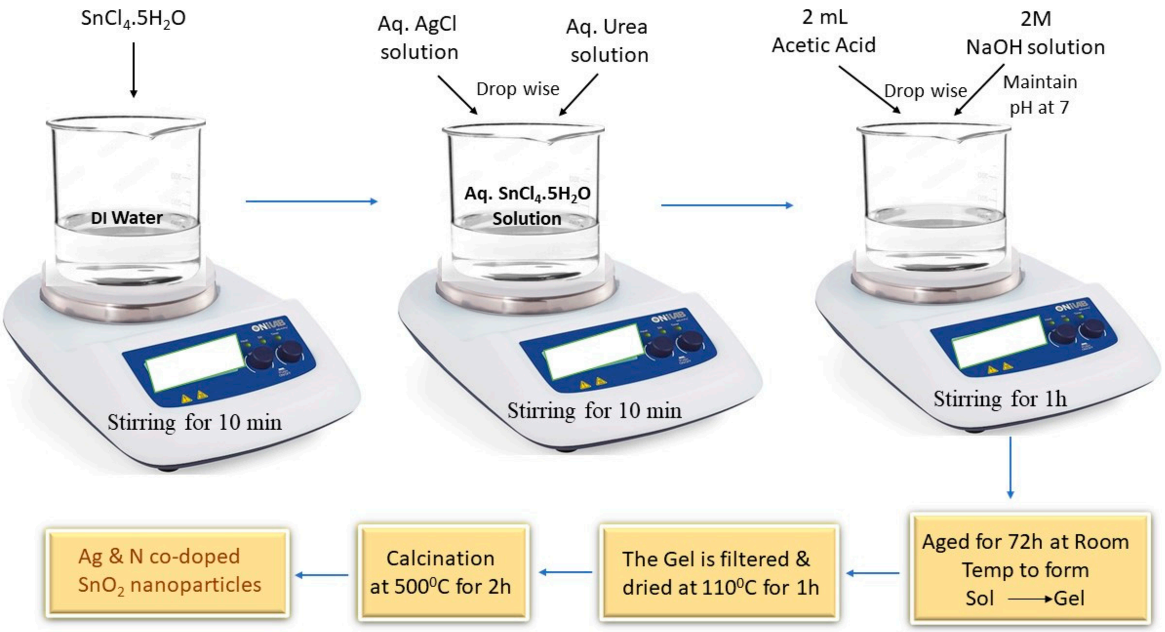

Figure S1. Block diagram for the synthesis of Ag-N-SnO_2 photocatalysts.

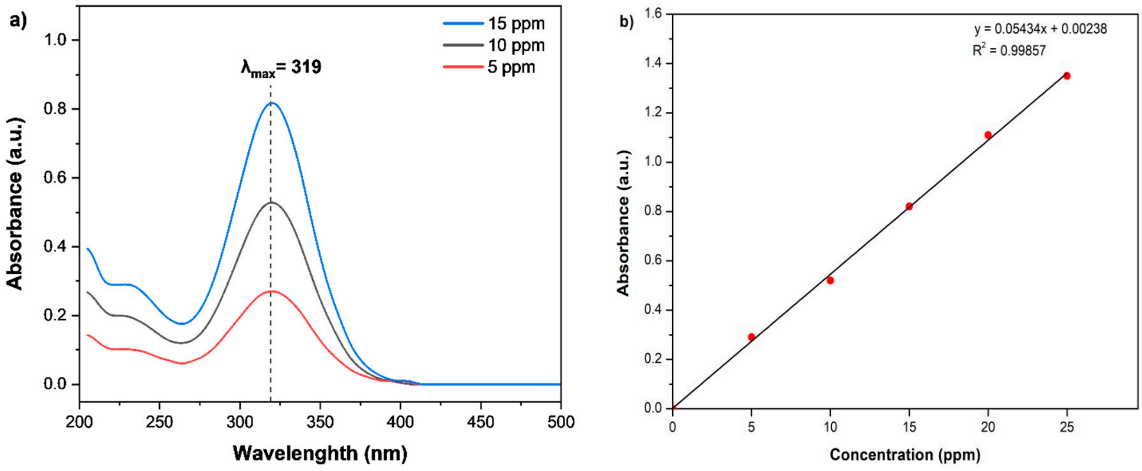

Figure S2. (a) UV-visible spectrum of standard metronidazole (MNZ) solutions at different concentrations and (b) calibration curve of MNZ.

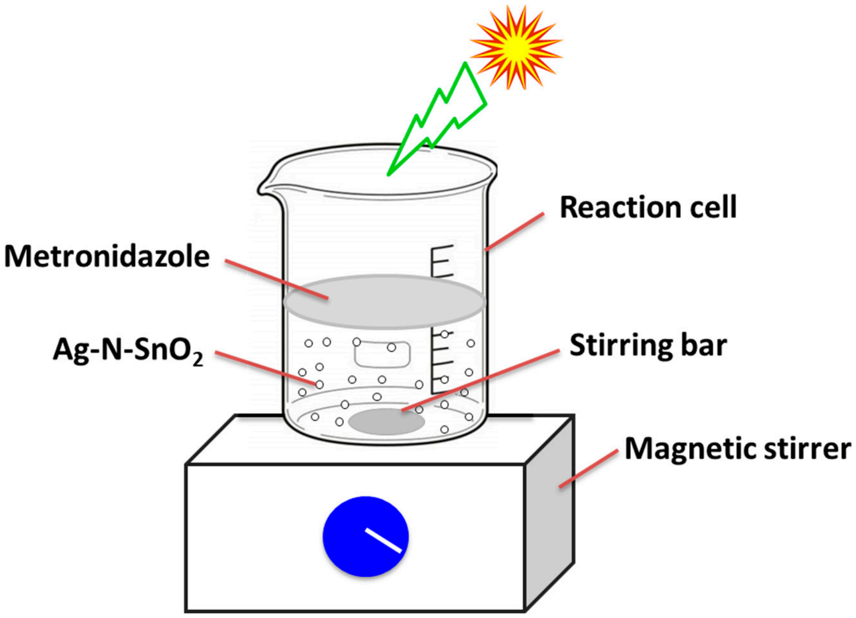

Figure S3. Diagram of the photocatalytic MNZ degradation reactor.

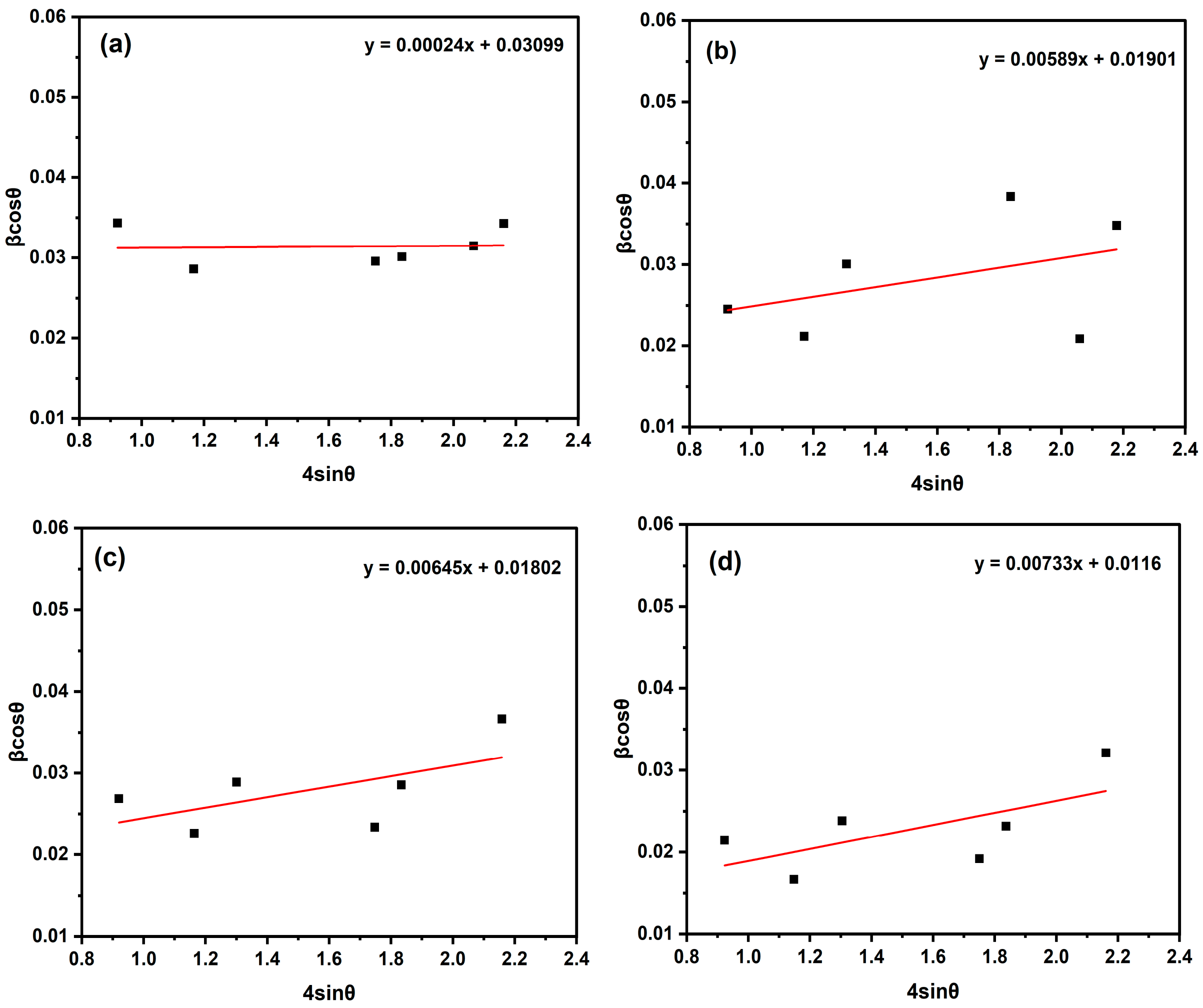

Figure S4. Williamson-Hall plots of (a) SnO_2 (b) Ag-SnO_2 (c) N-SnO_2 (d) Ag-N-SnO_2.

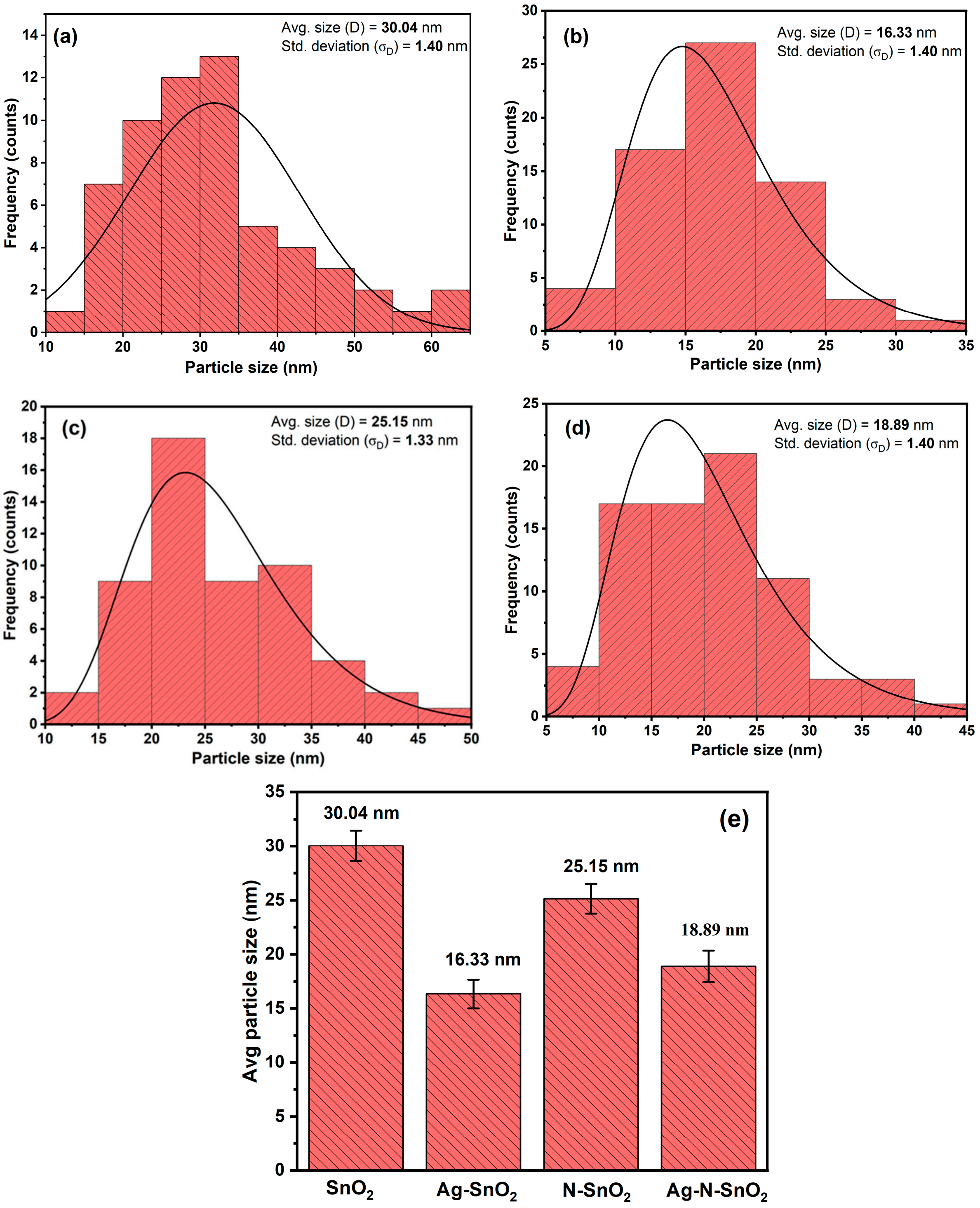

Figure S5. Particle size distribution histogram of (a) SnO_2, (b) Ag-SnO_2, (c) N-SnO_2, and (d) Ag-N-SnO_2, and (e) average particle size of the photocatalysts.

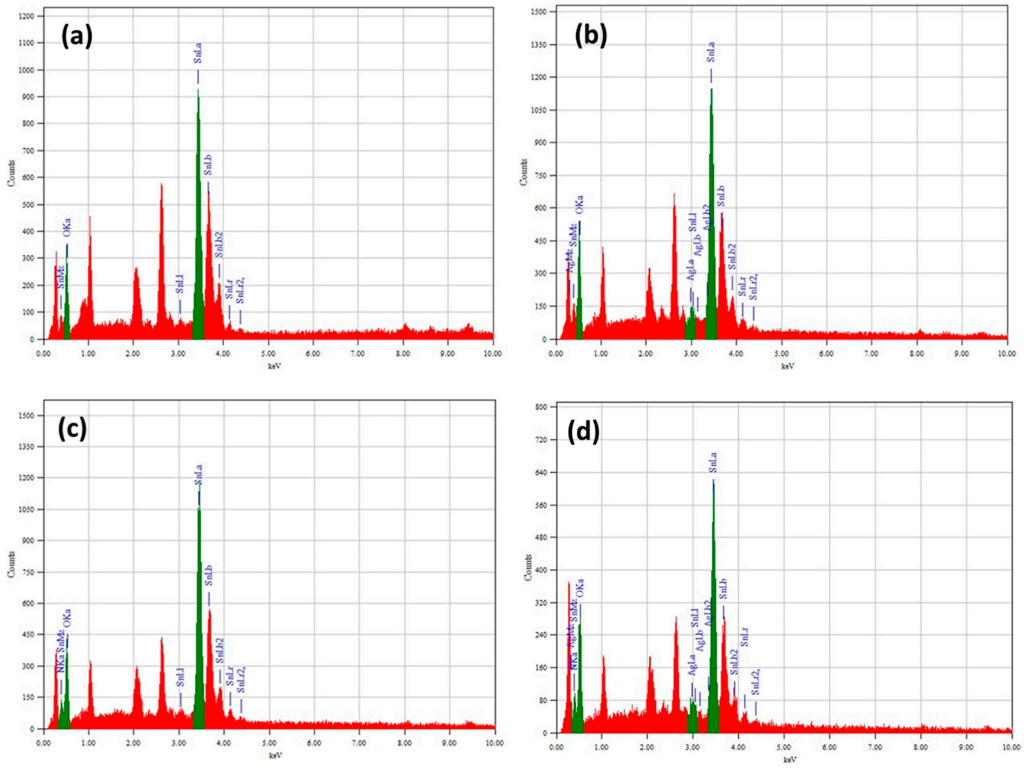

Figure S6. EDS pattern of (a) SnO_2, (b) Ag-SnO_2, (c) N-SnO_2, and (d) Ag-N-SnO_2.

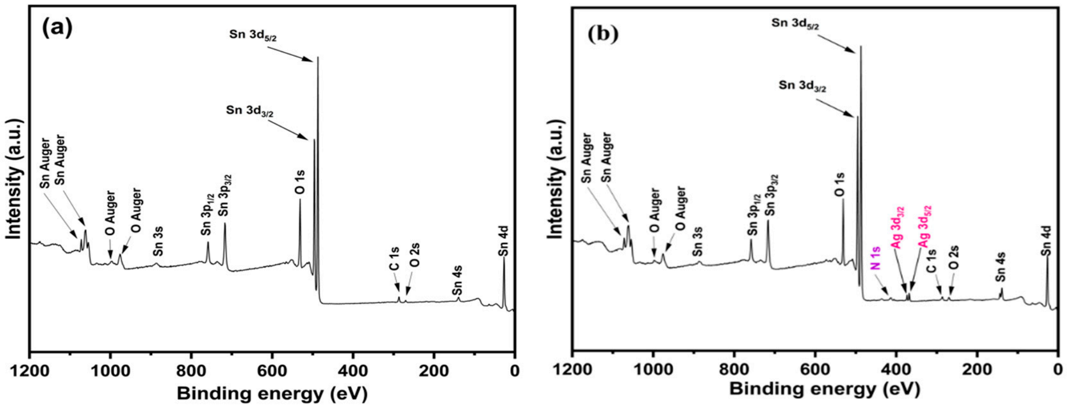

Figure S7. XPS survey spectra of (a) SnO_2 and (b) Ag-N-SnO_2.

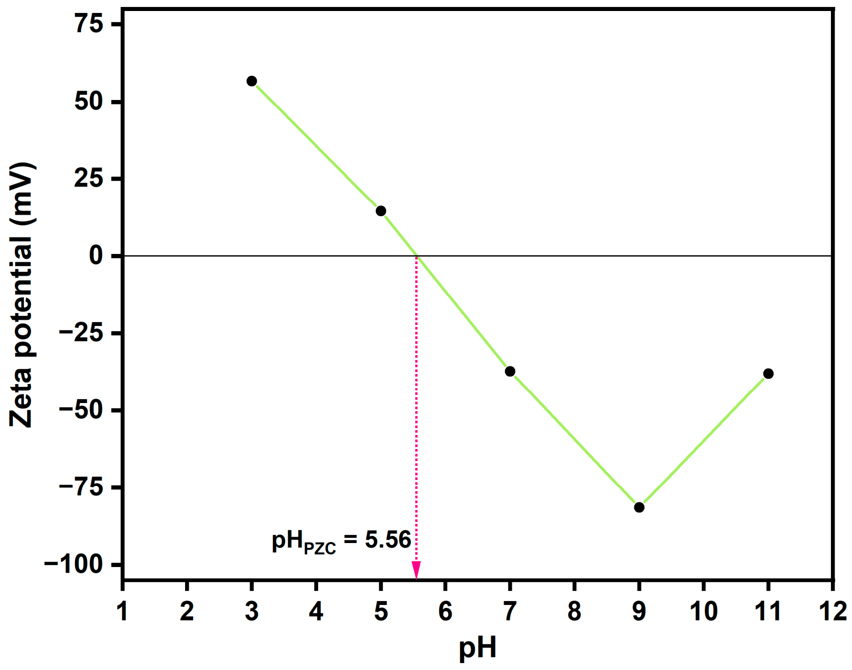

Figure S8. Zeta potential measurements of Ag-N-SnO_2 at different pH values.

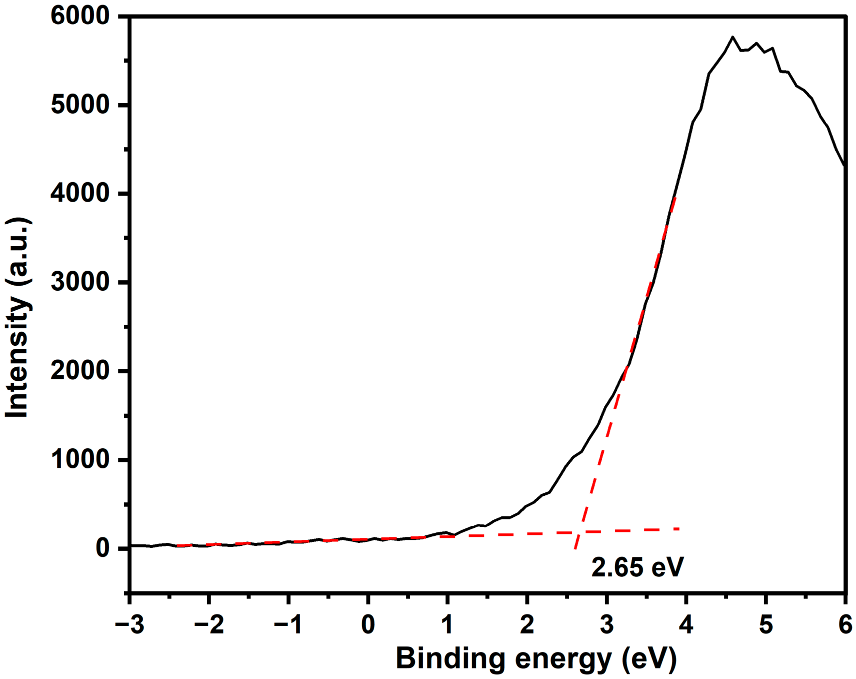

Figure S9. XPS valence band spectra of Ag-N-SnO_2.

Table 11. Elemental analysis of SnO_2, Ag-SnO_2, N-SnO_2, and Ag-N-SnO_2 from EDS.

Photocatalysts	Elements	Mass %	Atom %
SnO_2	Tin (Sn)	69.93	23.86
	Oxygen (O)	30.07	76.14
Ag-SnO_2	Tin (Sn)	63.46	19.99
	Oxygen (O)	33.85	79.08
	Silver (Ag)	2.69	0.93
N-SnO_2	Tin (Sn)	66.92	21.23
	Oxygen (O)	30.25	71.18
	Nitrogen (N)	2.83	7.60
Ag-N-SnO_2	Tin (Sn)	58.32	16.58
	Oxygen (O)	35.14	74.10
	Silver (Ag)	3.07	0.96
	Nitrogen (N)	3.47	8.36

Table S2: Atomic percentage and binding energies of different elements of Ag-N-SnO_2 from XPS.

Elements	Binding energy (eV)	Atom %
O 1s	531.06	56.71
Sn 3d	487.00	23.50
Ag 3d	367.78	1.17
N 1s	405.36	1.69
C 1s	286.18	11.07
Cl 2p	199.43	5.87
